# Investigating the Molecular Basis of the Aggregation Propensity of the Pathological D76N Mutant of Beta-2 Microglobulin: Role of the Denatured State

**DOI:** 10.3390/ijms20020396

**Published:** 2019-01-18

**Authors:** Lorenzo Visconti, Francesca Malagrinò, Luca Broggini, Chiara Maria Giulia De Luca, Fabio Moda, Stefano Gianni, Stefano Ricagno, Angelo Toto

**Affiliations:** 1 Istituto Pasteur-Fondazione Cenci Bolognetti, Dipartimento di Scienze Biochimiche “A. Rossi Fanelli” and Istituto di Biologia e Patologia Molecolari del CNR, Sapienza Università di Roma, 00185 Rome, Italy; lorenzo.visconti@uniroma1.it (L.V.); francesca.malagrino@uniroma1.it (F.M.); Angelo.Toto@uniroma1.it (A.T.); 2 Dipartimento di Bioscienze, Università degli Studi di Milano, 20133 Milano, Italy; luca.broggini@unimi.it; 3 Fondazione IRCCS Istituto Neurologico Carlo Besta, Divisione di Neurologia 5-Neuropatologia, 20133 Milano, Italy; chiara.deluca@istituto-besta.it (C.M.G.D.L.); Fabio.Moda@istituto-besta.it (F.M.)

**Keywords:** protein stability, denatured state, protein aggregation

## Abstract

Beta-2 microglobulin (β2m) is a protein responsible for a pathologic condition, known as dialysis-related amyloidosis (DRA), caused by its aggregation and subsequent amyloid formation. A naturally occurring mutation of β2m, D76N, presents a higher amyloidogenic propensity compared to the wild type counterpart. Since the three-dimensional structure of the protein is essentially unaffected by the mutation, the increased aggregation propensity of D76N has been generally ascribed to its lower thermodynamic stability and increased dynamics. In this study we compare the equilibrium unfolding and the aggregation propensity of wild type β2m and D76N variant at different experimental conditions. Our data revealed a surprising effect of the D76N mutation in the residual structure of the denatured state, which appears less compact than that of the wild type protein. A careful investigation of the structural malleability of the denatured state of wild type β2m and D76N pinpoint a clear role of the denatured state in triggering the amyloidogenic propensity of the protein. The experimental results are discussed in the light of the previous work on β2m and its role in disease.

## 1. Introduction

Several types of human diseases, spanning from Alzheimer’s disease to systemic amyloidosis, are caused by the incorrect folding of proteins [1]. A common factor of such pathological conditions lies in the accumulation of toxic aggregates [2]. Frequently, these aggregates are characterized by a specific type of highly ordered and stable structures, known as amyloid fibers, which are characterized by a conserved cross-beta structure and that can be identified with specific assays [3,4,5].

Beta-2 microglobulin (β2m) is a 99 residue protein forming part of the major histocompatibility complex class I (MHC-I). The aggregation and subsequent amyloid formation of β2m has been associated with a pathological condition known as dialysis-related amyloidosis (DRA) [6,7]. In fact, in dialyzed patients, there is an abnormally high concentration of β2m in blood. Such concentration of β2m exposes DRA patients to the risk of β2m amyloid deposition, a process that occurs mainly in joints and bones [8]. Intriguingly, β2m aggregation depends on several molecular properties and its initiation requires at least partial unfolding of the native state [9,10]; β2m aggregation propensity correlates well with thermodynamic stability [11] but recently an important role for protein dynamics in determining amyloidogenicity was also reported [12,13]. Thus, this protein system represents a suitable candidate to investigate the links between protein folding, misfolding, and pathology.

The first naturally occurring mutant of β2m was identified in 2012 [14]. This variant is much more aggressively amyloidogenic in vitro and in vivo compared to wt β2m and corresponds to a mutation at position 76, where a D is mutated to N. Biophysical characterization of the D76N variant in vitro demonstrated that the mutation triggers remarkable effects by decreasing the thermodynamic stability of the protein and increasing its propensity to form amyloids [14,15].

**Figure 1:**
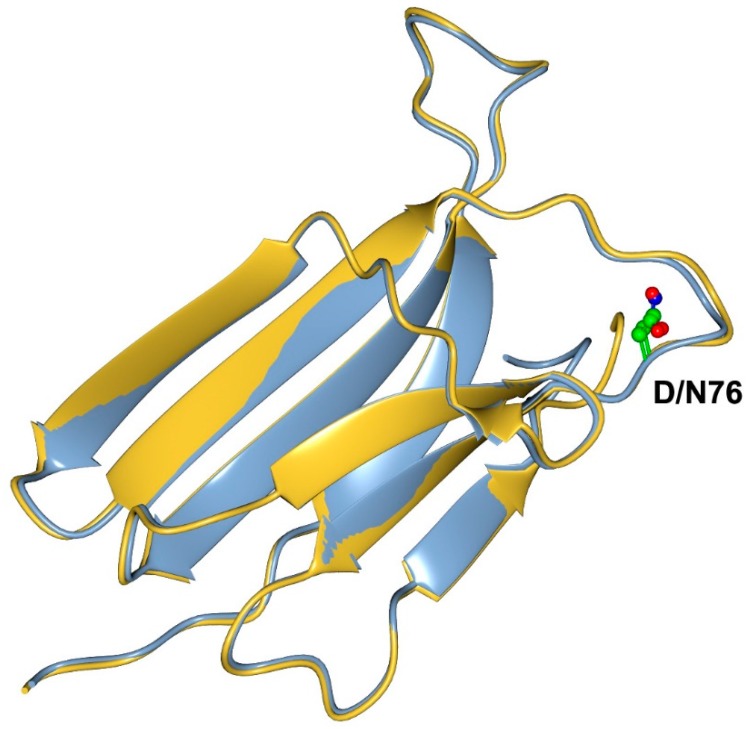
Three-dimensional structure of the D76N mutant (blue) in comparison to that of the wild type protein (yellow). Residue 76 is shown in ball and stick representation. It is evident that the native states of the two proteins are perfectly superimposable.

A structural analysis of the D76N mutant compared to the wt β2m highlighted an interesting conundrum (Figure 1). 

In fact, despite the dramatic effect of the mutation, residue 76 is located on a loop exposed to the solvent and the structure of the mutant is essentially identical to that of the wild type [14]. Thus, a comprehensive analysis of the biophysical and structural data available on D76N demonstrated that the destabilizing effect of the mutation is due to a complex effect involving an increased protein dynamics promoting the accumulation of a high-energy aggregation prone species [16].

Since the effect of the D76N mutation cannot be solely ascribed to direct effects on the native state, here we provide a characterization of the equilibrium folding behavior of the mutant in comparison to that of wt β2m. Data reveal that the destabilizing effect of the mutation is at least in part due to a change in its denatured state, which appears less compact than that of the wild type protein. Furthermore, by studying the equilibrium denaturation at different experimental conditions, we investigate the links between the compactness of the denatured state and the aggregation propensity of the protein. Our data are briefly discussed in the context of previous work on β2m.

## 2. Results

### 2.1. Equilibrium Unfolding Experiments

**Figure 2:**
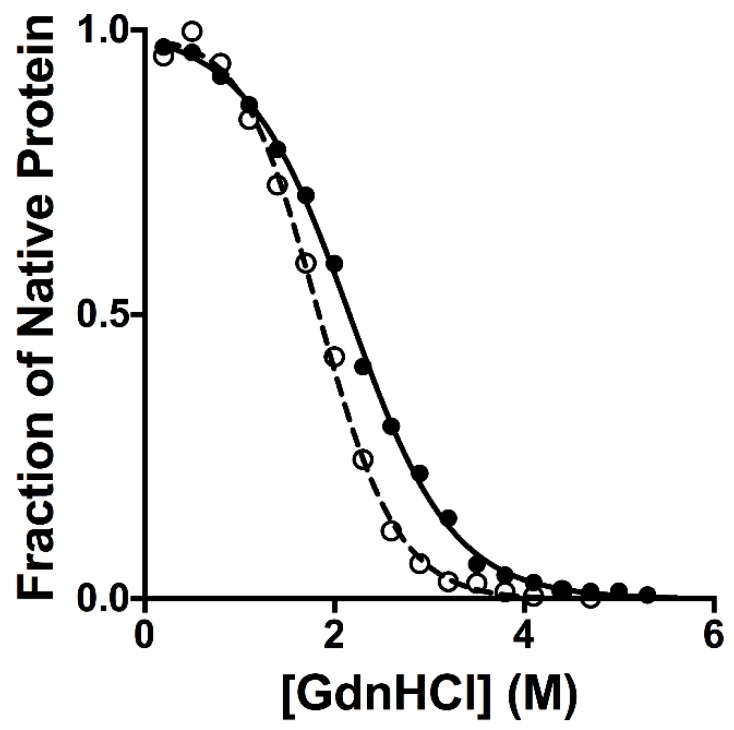
Equilibrium denaturations of wild type (full circles) and D76N variant (empty circles) performed in Hepes 50 mM pH 7.0 at 25 °C. The full line (for wild type) and the broken line (for D76N) are the best fit of an equation describing a two state unfolding mechanism. It is evident that the mutant displays a higher cooperativity compared to that of the wild type protein.

The thermodynamic stabilities of β2m wt and D76N variant were previously explored using thermal denaturations [16]. These studies showed a destabilization of the mutant, as mirrored by a ∆T_m_ of 10 K. In order to further compare the equilibrium unfolding of β2m wt and D76N, we conducted GdnHCl-induced equilibrium denaturation experiments. Protein denaturation was monitored by measuring the fluorescence emission of the two tryptophan residues in position 60 and 95 at different concentrations of GdnHCl. In both cases, observed fluorescence was consistent with a simple sigmoidal transition, characteristic of two-state folding. The robustness of the two-state equilibrium transition for both β2m wt and D76N was further confirmed by fitting globally the fluorescence profiles obtained at different wavelengths with shared thermodynamic parameters. The dependence of the normalized observed fluorescence signal at 330 nm versus the concentration of denaturant for wt and D76N is reported in Figure 2. It is evident that, whilst the mutant unfolds at lower concentrations of GdnHCl, the apparent cooperativity of the transition is affected by the mutation, with an increase of cooperativity for D76N as compared to that of the wild type protein.

A powerful parameter to infer the mechanism of folding single domain proteins is the m_D-N_ value, defined as ∂∆G/∂[denaturant], which is the quantitative measurement of the cooperativity of the transition. In fact, the m_D-N_ value is correlated to the change in the accessible surface area to the solvent upon unfolding [17] and allows therefore detecting indirectly the overall structural transition occurring between the native and denatured states. The m_D-N_ values obtained for β2m wt and D76N were 1.08 ± 0.02 kcal mol^−1^ M^−1^ and 1.45 ± 0.04 kcal mol^−1^ M^−1^, respectively, highlighting an effect of the mutation on the cooperativity of the unfolding reaction. Since it was previously established that β2m wt and D76N share a nearly identical native state [14], a decreased change in the accessible surface area upon denaturation can be ascribed to a more compact denatured state, highlighting the presence of a residual structure in the denatured state. Thus, on the basis of the comparison between the observed m_D-N_ values for β2m wt and D76N it may be concluded that the mutation leads to an expansion of the polypeptide chain in the denatured state. 

**Figure 3:**
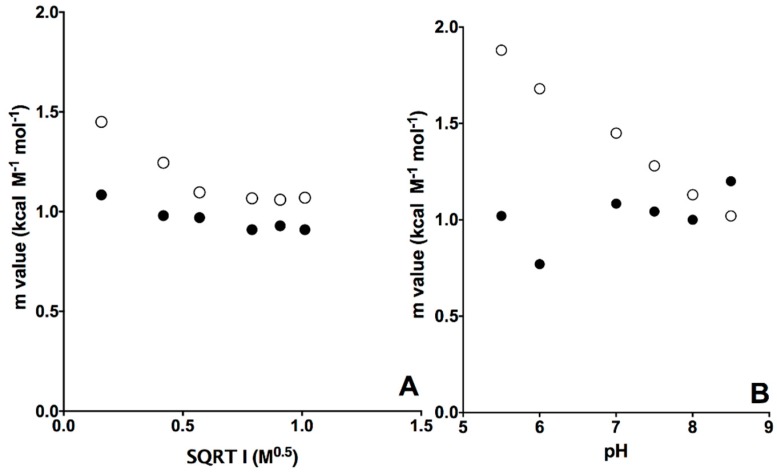
Dependence of m_D-N_ values calculated at different ionic strengths (panel **A**) and pH (panel **B**) for wt (full circles) and D76N variant (empty circles).

Since the analysis of m_D-N_ values represents a signature of the residual structure in denatured states, by challenging the system at different experimental conditions, it is possible to monitor the structural malleability of the denatured state and therefore to characterize its shifts along the reaction coordinate. Thus, we resorted to perform equilibrium unfolding experiments at different experimental conditions, i.e., by varying the pH and ionic strength. The dependence of calculated m_D-N_ versus pH and the square root of the ionic strength at pH 7.0 for wt and D76N is reported in Figure 3 and the associated folding parameters are listed in Table 1. 

Inspection of the experimental data reveals that while the m_D-N_ value of wild type β2m is essentially insensitive to pH and ionic strength, for the D76N variant there is an evident decrease of the m_D-N_ value with increasing pH and ionic strength. Overall, these observations indicate the denatured state of wild type β2m to be characterized by a malleable residual structure. Such structure is perturbed in D76, but becomes more compact as the pH and ionic strength of the solution increase. 

### 2.2. Aggregation Essays

In a recent comprehensive biophysical and structural work, it was proposed that the aggregation properties of D76N to be ascribed to the population of an aggregation-prone, highly dynamic, species, which is more compact and less aggregation prone in the wild type protein [18]. On the light on the equilibrium folding transitions described above, it is tentative to speculate that among these aggregation-prone species the denatured state of the protein may play a role, being more expanded for D76N than that of the wild type protein. A possible test to verify this hypothesis would be represented by the comparison of the aggregation propensity of D76N as a function of ionic strength and pH and the dependence of its associated unfolding m_D-N_ values. 

**Figure 4:**
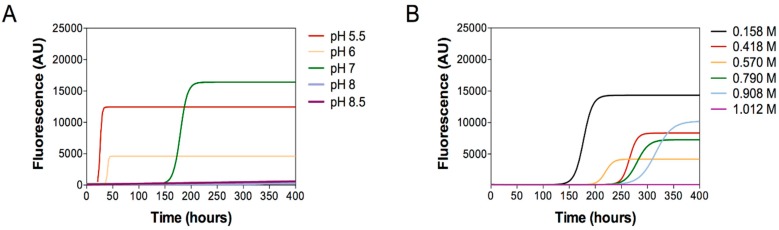
Aggregation experiments of D76N as function of pH (panel **A**) and of ionic strength (panel **B**). Aggregation was followed by monitoring thioflavin T (ThT) fluorescence. The mean values of the three independent experiments subjected to nonlinear regression analysis, using Boltzmann sigmoidal equation, are reported.

To this aim, we performed aggregation experiments of D76N (40 µM) under continuous shaking at 37 °C under the same conditions of pH and of ionic strength, which are relevant in modifying the compactness of D76N unfolded state. The aggregation kinetics were monitored by thioflavin T showing that the aggregation lag time is increased as pH and ionic strength increase (Figure 4A,B, respectively, where the mean values of the three independent experiments subjected to nonlinear regression analysis, using Boltzmann sigmoidal equation, are reported). ThT fluorescence tends to be more intense in samples, which quickly aggregate suggesting a more abundant amyloid formation under certain conditions; however, fluorescence intensity strongly depends on experimental conditions and should be considered with care.

In summary these results clearly showed that D76N aggregation propensity is enhanced by low pH (Figure 4A) and by low ionic strength (Figure 4B). In contrast, high pH conditions or high salt concentrations delay or totally abrogate protein aggregation, even after 400 h of reaction.

## 3. Discussion

The denatured state of proteins has historically received considerably less consideration than the native state. In fact, the latter exerts all the biological functions and characterizing its structure is typically a key step in understanding them. Additionally, addressing denatured states is particularly difficult as they are elusive to the classical structural biology techniques and may be populated only under certain conditions.

In this context, it is important to distinguish between the unfolded state, the highly disordered conformation that may be populated in the presence of denaturant, and the denatured state, which retains a considerable amount of residual structure and represents a transient on-pathway species accumulating only at very low denaturant concentrations (or in the absence of it) [19,20,21]. Whilst the unfolded state often corresponds to an expanded random coil conformation, the residual structure of the denatured state is critical in sculpting the folding pathways of proteins, as well as in committing the protein to a specific topology [22,23,24,25,26,27].

The destabilization effects of the pathological D76N mutant of β2m represents a paradigmatic example on how the structural studies on the sole native state do not provide a full picture on protein stability, being the native state of both variants essentially identical [14,15]. From this angle, the experiments reported in this work are particularly informative as they point out a crucial role to the destabilizing effects in the denatured state induced by the D76N mutation. In fact, the experimental data suggest a scenario whereby the apparent ∆∆G observed in thermal denaturation experiments is most likely to arise from the expansion, and consequent increase in free energy, of the denatured state rather than changes in the native conformation.

Previous studies on the amyloidogenic properties of β2m have demonstrated that aggregation might occur only when the protein transiently escapes the thermodynamic well of its native state [9,10]. This finding would suggest that in the case of D76N, there should be a correlation between thermodynamic stability and aggregation propensity. A comparison between the equilibrium unfolding and aggregation data obtained at different pH and ionic strengths, however, shows a much more complex scenario. In fact, while the protein is mildly destabilized at high ionic strengths, it is evident that its thermodynamic stability is essentially insensitive to pH between 8.5 and 7. However, under such conditions D76N displays completely different kinetics of amyloid aggregation. Thus, we conclude that the strong correlation of the amyloidogenic propensity the thermodynamic stability underlies a strong contribution from the overall structure of the denatured state. Increasing amount of data from solid state NMR and Cryo-EM [28,29,30,31] show that proteins with an ordered native fold such as β2m and immunoglobulin light chains display a structural organization in fibrils, which is completely different from the native fold. This observation directly suggests that these proteins need to totally unfold during the aggregation pathway.

The experiments reported in this work indicate that the denatured state has a malleable structure that is perturbed from changes in experimental conditions. The overall compactness of such state correlates with the aggregation propensity of D76N. Overall, our work represents a direct demonstration on the key role of denatured states in dictating protein aggregation and reinforces the importance of their characterization. 

## 4. Materials and Methods

### 4.1. Protein Expression and Purification

Recombinant wt and D76N β2m were expressed and purified as previously reported [12].

### 4.2. Equilibrium Experiments

Equilibrium unfolding experiments on wt and D76N β2m were performed at 25 °C using a Fluoromax single photon counting spectrofluorometer (Jobin-Yvon; Edison, NJ, USA) and a quartz cuvette with a path length of 1 cm. The native protein, at a constant concentration of 2 µM, was mixed with increasing concentrations of the denaturant agent guanidium chloride (GdnHCl), and the intrinsic tryptophan emission of the residues in position 60 and 95 was measured by excitation at 280 nm and record of emission spectra between 300 and 400 nm. Fluorescence equilibrium experiments at different pH were carried out using 50 mM sodium acetate pH 5.5, 50 mM BisTris pH 6.0 pH 6.5, 50 mM HEPES pH 7.0, 50 mM TrisHCl pH 7.5, pH 8.0, and pH 8.5 as buffers. In equilibrium denaturations at different ionic strength conditions, the buffer used was 50 mM HEPES pH 7.0 with different NaCl concentrations (0, 150 mM, 300 mM, 600 mM, 800 mM, and 1000 mM).

Data were fitted using the following equation,

$$Y_{obs}=\left(Y_{N}+Y_{D}\right)\frac{e^{m_{D-N}\left(\left[GdnHCl\right]-{\left[GdnHCl\right]}_{1/2}\right)}}{1+e^{m_{D-N}\left(\left[GdnHCl\right]-{\left[GdnHCl\right]}_{1/2}\right)}}$$

### 4.3. Aggregation Assays 

Aggregation assays of D76N β2m at different pH (5.5, 6, 7, 8, and 8.5) were performed in reaction mix containing D76N β2m (40 µM) and ThT (10 µM).

Aggregation assays of D76N β2m at different concentration of NaCl (0, 150 mM, 300 mM, 600 mM, 800 mM, and 1000 mM) were performed in 25 mM Na phosphate pH 7.4 containing D76N β2m (40 µM) and ThT (10 µM).

All reactions were performed in triplicate using black, clear-bottom, 96-well microplates. After sealing, the plate was incubated in a FLUOstar OPTIMA reader (BMG Labtech, Germany) at 37 °C, over a period of 400 h with continuous shaking (600 rpm, single orbital). The ThT fluorescence values are expressed in arbitrary units (AU) and were taken every hour using 450 ± 10 nm (excitation) and 480 ± 10 nm (emission) wavelengths, with a bottom read and a gain of 1000. The mean ThT fluorescence values per sample were plotted against time (hours).

The mean ThT fluorescence values of the three replicates per sample were plotted against time (hours) and the obtained curves were subjected to nonlinear regression analysis, using Boltzmann sigmoidal equation.

## Figures and Tables

**Table 1:** Equilibrium folding parameters of wild type and D76N β2m.

	**β2m Wild Type**		**β2m D76N**	
**IS^1/2^ (M^1/2^)**	**[GdnHCl]_1/2_ (M)**	**m_value_ (kcal M^−1^ mol^−1^)**	**[GdnHCl]_1/2_ (M)**	**m_value_ (kcal M^−1^ mol^−1^)**
0.16	2.12 ± 0.07	1.08 ± 0.03	1.82 ± 0.02	1.45 ± 0.04
0.42	2.07 ± 0.09	0.98 ± 0.03	2.13 ± 0.02	1.25 ± 0.03
0.57	2.02 ± 0.06	0.97 ± 0.05	1.95 ± 0.07	1.10 ± 0.03
0.79	2.12 ± 0.06	1.02 ± 0.02	1.79 ± 0.03	1.07 ± 0.03
0.91	2.21 ± 0.06	0.93 ± 0.01	2.03 ± 0.02	1.06 ± 0.04
1.01	1.61 ± 0.16	0.91 ± 0.06	1.42 ± 0.06	1.06 ± 0.06
	**β2m Wild Type**		**β2m D76N**	
**pH**	**[GdnHCl]_1/2_ (M)**	**m_value_ (kcal M^−1^ mol^−1^)**	**[GdnHCl]_1/2_ (M)**	**m_value_ (kcal M^−1^ mol^−1^)**
5.5	<1	1.02 ± 0.03	1.22 ± 0.02	1.88 ± 0.04
6	1.40 ± 0.16	0.77 ± 0.04	1.74 ± 0.02	1.74 ± 0.05
7	2.13 ± 0.07	1.08 ± 0.03	1.81 ± 0.02	1.45 ± 0.04
7.5	2.09 ± 0.08	1.04 ± 0.03	2.36 ± 0.02	1.28 ± 0.03
8	2.04 ± 0.09	1.00 ± 0.03	1.77 ± 0.05	1.12 ± 0.06
8.5	1.95 ± 0.04	1.23 ± 0.01	1.79 ± 0.03	1.02 ± 0.02
